# DP2 receptor activity sensor suited for antagonist screening and measurement of receptor dynamics in real-time

**DOI:** 10.1038/s41598-024-58410-2

**Published:** 2024-04-08

**Authors:** Michael Kurz, Michaela Ulrich, Alwina Bittner, Moritz Bünemann

**Affiliations:** Faculty of Pharmacy, Institute for Pharmacology and Clinical Pharmacy, Philipps-University Marburg, Marburg, Germany

**Keywords:** Drug screening, Pharmacology, Receptor pharmacology

## Abstract

The DP2 receptor is a G-protein coupled receptor involved in allergic inflammation and is the target of recently developed antagonists already being tested in clinics. To get insights into DP2 receptor dynamics and to study its pharmacology on the level of the receptor, we constructed a fluorescence resonance energy transfer-based conformation sensor. The sensor reflects the selectivity profile of the DP2 receptor-wt and is suited for screening of agonists and antagonists due to its robust response. Furthermore, the sensor enables the direct measurement of DP2 receptor dynamics in real-time and revealed markedly distinct on- and off-rates of prostaglandin D_2_ between DP2 and DP1 receptors, suggesting a different mechanism of ligand receptor interaction.

## Introduction

The DP2 receptor/CRTh2 (chemoattractant receptor-homologous molecule expressed on Th2 cells) is a member of the G-protein coupled receptor class. It is named after its endogenous agonist prostaglandin (PG) D_2_, but it is more closely related to chemoattractant receptors than other prostanoid receptors^1^. DP2 receptor is primarily expressed in T helper type 2 (Th2) cells, basophils, eosinophils, group 2 innate lymphoid cells (ILC2) and type-2 cytokine-secreting CD8^+^CRTH2^+^ (Tc2) cells^2–4^. The expression of the DP2 receptor is not limited to immune cells: high RNA levels were detected in the stomach, small intestine, heart and thymus^5^. DP2 receptor signaling could be linked to allergic inflammation and DP2 receptor antagonism has been identified as a strategy to treat allergic diseases in humans^6^. Many DP2 receptor antagonists have been developed and tested in clinics, such as AZD1981, fevipiprant and the dual DP2 receptor and the thromboxane receptor antagonist ramatroban which is licensed for the treatment of allergic rhinitis^6–9^. A recent case report suggested a potential role for ramatroban in the treatment of COVID-19^10^. Among prostaglandins, PGD_2_ is the PG most abundant in the brain and is involved in the regulation of nociception, temperature and sleep^11^. In respect to sequence homology the DP2 receptor differs the most from the other prostanoid receptors, which might explain differences in structure, ligand entry and binding mode^12,13^. It is a promiscuous receptor activated not only by PGD_2_ but also by the PGD_2_ metabolite 13,14-dihydro-15-keto PGD_2_ (DK PGD_2_) and even the non-prostanoid related COX inhibitor indomethacin^1,5^. The aforementioned ligands have been investigated in different studies on aspects such as binding, signaling on the level of second messengers and in functional readouts such as chemotaxis^2,5,14^. As it is difficult to directly compare agonist efficacies derived from downstream assays due to signal amplification and non-linearity of signal changes in relation to changes in the receptor activity, we set out to measure ligand-induced DP2 receptor activity at the level of the receptor itself. Therefore, we constructed a Förster resonance energy transfer (FRET) based conformation sensor of the human DP2 receptor. Our sensor allowed for measurements in both single and multiple cell FRET recordings and was suited for the characterization of antagonists.

## Results

### Successful construction of a FRET-based human DP2 receptor conformation sensor

**Figure 1:**
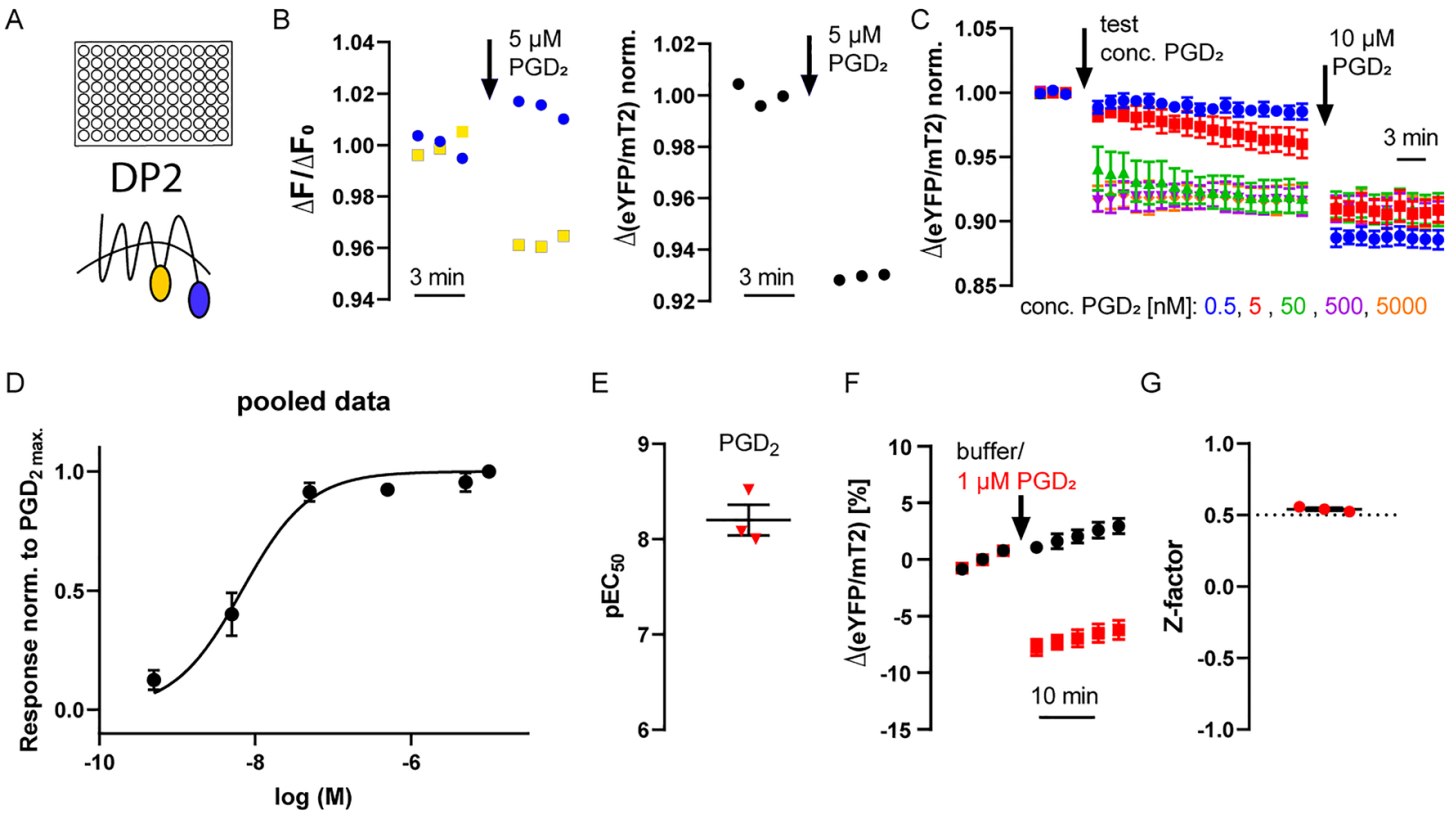
Human DP2 receptor conformation sensor suited for measurements in microtiter plates. (**A**) FRET measurements were performed in 96-well plates containing HEK293 cells stably expressing the DP2 receptor sensor using a Spark 20 M (Tecan) plate reader. (**B**) Left: depicted is a representative example of the time course of mTurq2 and eYFP emission upon excitation at 430 nm in a single well (corrected for buffer wells, normalized to the three basal cycles). Right: the corresponding alteration in the emission ratio was plotted as Δ(eYFP/mTurq2) of a representative example single well measurement upon addition of 5 µM PGD_2_. (**C**) Averaged Δ(eYFP/mTurq2) signals were plotted from experiments similar as described in (**B**) where a test concentration of PGD_2_ was applied at cycle 3 followed by reference concentration of 10 µM PGD_2_ at cycle 20 (final concentration). (**D**) Each agonist-evoked amplitude of the tested PGD_2_ concentrations was normalized to the amplitude of the reference concentration. The panel shows the mean ± SEM of the DP2 receptor sensor activation from all experiments and a curve fit to the pooled data (for illustration purposes only). (**E**) The panel shows pEC_50_ for three individual experiments. (**F**) Δ (eYFP/mTurq2) changes in percent of an example plate used to determine the Z-factor of DP2 receptor sensor are plotted over time. Control wells are shown in black (buffer application) and sample wells in red (PGD_2_ application), n = 30 per group. (**G**) Z-factor values of three independent DP2 receptor sensor experiments are plotted. Individual experiments are presented in Fig. 1S. (**C**–**G**) Data are presented as mean ± SEM.

The DP2 receptor FRET conformation sensor was constructed by inserting a fluorophore into the third intracellular loop (eYFP) and another to the C-terminus (mTurquoise2, mTurq2) analog to the previously described construction of TP receptor sensor and EP4 receptor sensor^15,16^. The receptor sensor construct was stably expressed in human embryonic kidney (HEK293) cells. We performed FRET measurements of multiple cells in 96 well plates on a plate reader (Fig. 1A). We observed an increase in cyan emission and a simultaneous decrease in yellow emission upon application of the endogenous agonist PGD_2_ indicating a change in FRET (Fig. 1B). The emission ratio of eYFP emission divided by mTurq2 emission was calculated and plotted (Fig. 1C). The effect of the PGD_2_ application on the emission ratio was concentration dependent. The data was normalized by the final concentration of 10 µM PGD_2_ and a concentration response curve could be fitted giving rise to a pEC_50_ value of 8.20 (95% CI 7.50–8.90; Fig. 1D,E). Next, we evaluated the suitability of our newly established assay for high throughput screenings. HEK293 cells stably expressing DP2 receptor sensor were seeded in microplates of a 96 well plate. The cells residing in control wells were treated with buffer, while sample wells were treated with a final concentration of 1 µM PGD_2_ (Fig. 1F; S1 Fig). The Z-factor value was determined to be 0.5412 ± 0.0096 (mean ± SEM), which corresponds to an excellent assay^17^, (Fig. 1F,G; S1 Fig).

### DP2 receptor sensor reflects the selectivity profile of the DP2 receptor-wt

**Figure 2:**
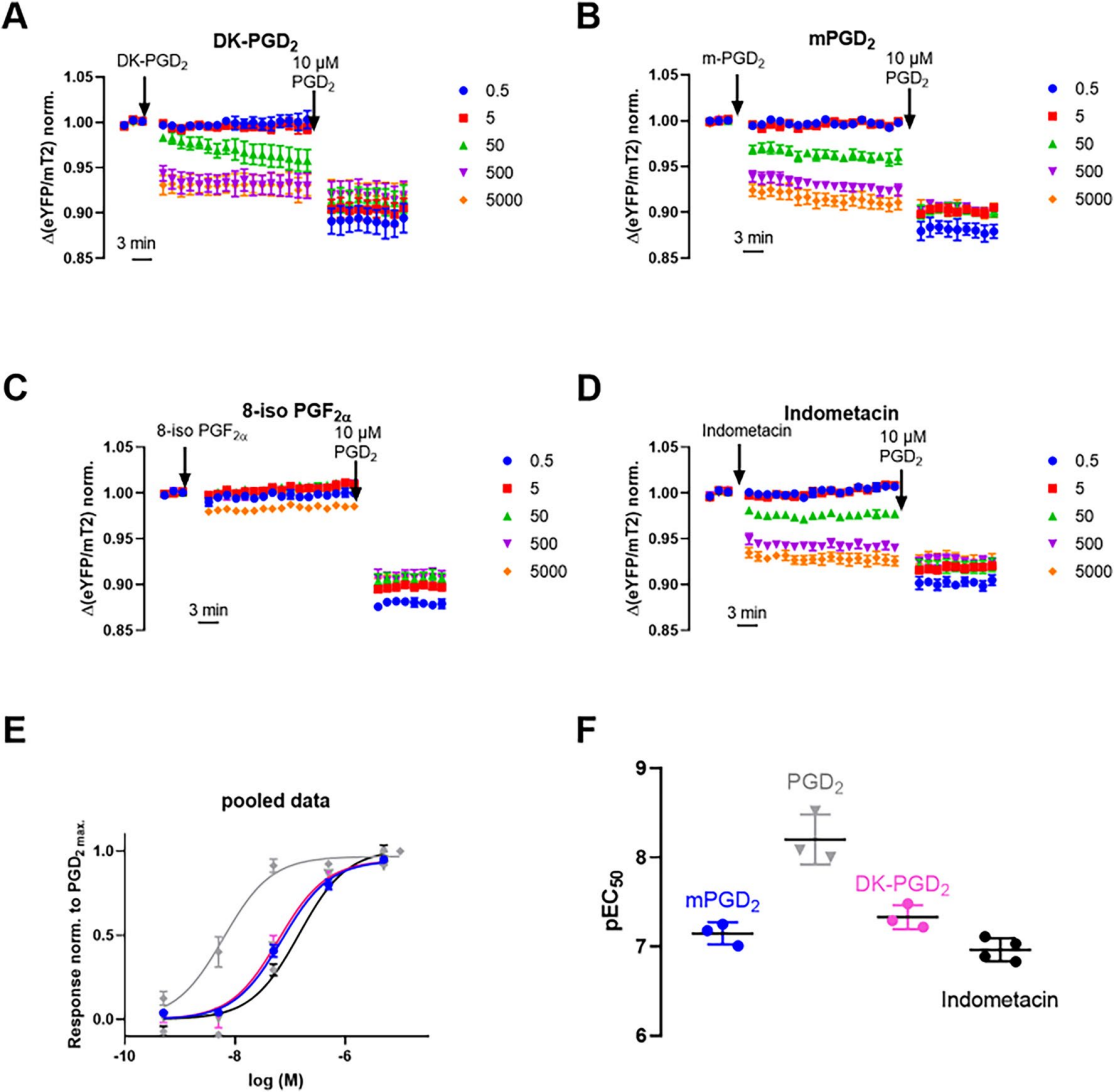
Effect of selected ligands on DP2 receptor activity. (**A**–**D**) Plotted are the averaged data of the effect of selected ligands on the DP2 receptor sensor activity. The measurements were performed analog to the measurements described in Fig. 1C. (**E**) Each agonist-evoked amplitude of the tested ligands was normalized to the amplitude of the reference concentration of PGD_2_ within the same well. The panels show the mean ± SEM of the DP2 receptor sensor activation from all experiments of the respective ligand and a curve fit to the pooled data (for illustration purposes only). PGD_2_ is depicted in gray, mPGD_2_ in blue, DK PGD_2_ in pink and indomethacin in black. The data for PGD_2_ were derived from Fig. 1 and are plotted for a better comparability. (**E**) Each agonist-evoked amplitude of the tested ligands was normalized to the amplitude of the reference concentration of 10 µM PGD_2_. The panel shows the mean ± SEM of the DP2 receptor sensor activation from all experiments and for demonstration purposes a curve fit to the pooled data. The panels in (**E**) shows the shows the pEC_50_ values for three to four individual experiments measured for each ligand, mean ± SEM.

We tested the effect on the activity of the DP2 receptor sensor of different arachidonic acid products and stable analogues with measurements, as described for Fig. 1C, normalizing all values to the response evoked by 10 µM PGD_2_ (Fig. 2, 2S Fig). 10 µM of either iloprost, the stable analog of prostacyclin, U-46619, the stable analog of PGH_2_, or PGE_2_ were tested. While iloprost showed only a minor, but statistically significant, activation of the DP2 receptor sensor compared to the PGD_2_ evoked response, the normalized responses caused by U-46619 and PGE_2_ were not significantly different from 0 (2S Fig: 6% ± 1%, 7% ± 3%, 15% ± 4%; mean ± SEM). This is in line with the literature, which reports pK_i_ values for these compounds at DP2 receptor-wt < 5.52^5,18^. The PGD_2_ metabolite DK PGD_2_ which only binds DP2 receptor, but not DP1 receptor^5^, showed an pEC_50_ value of 7.33 (95% CI 7.00–7.66) and an E_max_ value as observed for PGD_2_ (Fig. 2A,E,F; 2S Fig). For unknown reasons, this value is slightly right shifted compared to the literature pK_i_ values for DK PGD_2_ which ranged from 7.40 to 8.52^5,18–21^. 15(S)-15-methyl PGD_2_ showed a pEC_50_ value of 7.15 (95% CI 6.84–7.45) and an E_max_ value as observed for PGD_2_ (Fig. 2B,E,F; 2S Fig). The pEC_50_ value is comparable to the literature pK_i_ value of 7.47^5^. The dihomo-γ-linolenic acid product PGE_1_ only showed minor, but statistically significant, activation at 10 µM (2S Fig: 16% ± 3%; mean ± SEM). Isoprostanes are generated in vivo in humans, independent of the cyclooxygenase (COX), by the free radical-induced peroxidation of arachidonic acid and show a chiral inversion in the C8 position^22^. We tested 10 µM of 8-iso PGE_1_ and 8-iso PGE_2_ and 5 µM of 8-iso PGF_2α_ against PGD_2_. While 8-iso PGF_2α_ showed only a minor, but statistically significant, activation of the DP2 receptor sensor compared to the PGD_2_ evoked response, the normalized responses caused by 8-iso PGE_1_ and 8-iso PGE_2_ were not significantly different from 0 (Fig. 2D, 2S Fig; 15% ± 1%, 6% ± 2% and 11% ± 3%, mean ± SEM). Lastly, the COX inhibitor indomethacin showed a pEC_50_ value of 6.97 (95% CI 6.76–7.17, Fig. 2D–F) and an E_max_ value as observed for PGD_2_ (2S Fig), which fits within the range of reported pK_i_ values in the literature ranging from 5.72 to 7.60^5,7,9,18,19^.

### FRET-based conformation sensors reveal major differences between the PGD_2_ induced receptor activation and deactivation kinetics at DP1 and DP2 receptor

**Figure 3:**
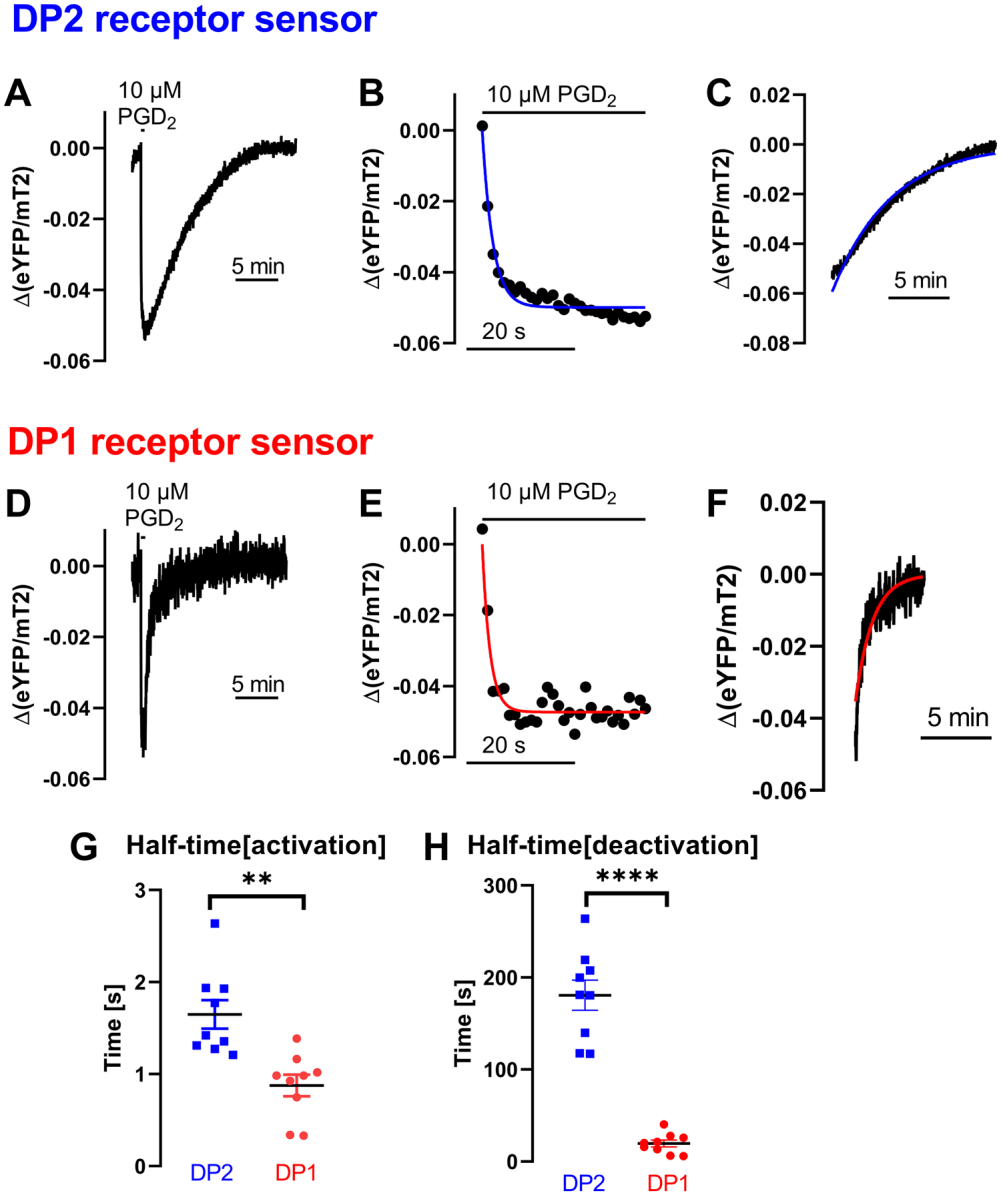
DP1 and DP2 receptor differ remarkably in their deactivation kinetics upon PGD_2_ withdrawal. We performed single cell FRET measurements of HEK293T cells transiently transfected with either DP2 or DP1 receptor sensor construct measured at an inverted fluorescence microscope under constant flow using a pressurized perfusion system. Depicted is an example trace of the time course of the DP2 receptor sensor activation induced upon PGD_2_ application and subsequent receptor deactivation upon agonist withdrawal (**A**). (**B**) shows the extract from the curve shown in (**A**) used to fit to a mono-exponential function to determine the kinetics of receptor activation. (**C**) shows the extract from the curve shown in (**A**) used to fit to a mono-exponential function to determine the kinetics of receptor deactivation. (**D**) shown is an example trace of the time course of DP1 receptor sensor activation and deactivation measured side by side to the measurements shown in (**A**). (**E**,**F**) show the respective extract of the curve shown in (**D**) used to calculate receptor (de)activation kinetics respectively. (**G**,**H**) Halftimes determined by fitting of individual experiments of (**G**) activation kinetics (*P* value 0.0011**, unpaired t test) and H) deactivation kinetics (*P* value < 0.0001****, unpaired t test with Welch's correction) are displayed as mean ± SEM (n = 8–9 per condition).

In recent years, different studies addressed the antagonist dissociation kinetics at the DP2 receptor, either using radio ligand binding directly or indirectly from functional [^35^S]-GTPγS assays^7,23–25^. FRET based GPCR conformation sensors are helpful tools to investigate receptor activation and deactivation kinetics and we were especially interested in the DP2 receptor kinetics evoked by the endogenous agonist PGD_2_. We measured the kinetics of DP2 receptor activation and deactivation applying 10 µM PGD_2_ in single cell recordings using a pressurized perfusion system with an inverted fluorescence microscope (Fig. 3A–C). The protocol started with 2 min of external buffer application, then we switched to 10 µM PGD_2_ for 30 s, followed by wash-out by the application of external buffer (Fig. 3A–C). The kinetics of receptor activation of PGD_2_ was determined to be 1.7 s ± 0.2 s (Fig. 3A,B,G; halftime, mean ± SEM). The kinetics of receptor deactivation upon PGD_2_ withdraw at DP2 receptor was determined to be 180.7 s ± 16.3 s (Fig. 3A,C,H; halftime, mean ± SEM). DP2 receptor and DP1 receptor share PGD_2_ as an endogenous agonist. To investigate and compare the kinetics of PGD_2_ at the DP1 receptor to those at the DP2 receptor we constructed a DP1 receptor sensor analog to the DP2 receptor sensor construct. The pEC_50_ of PGD_2_ at the DP1 receptor sensor was right shifted about factor six compared to the pEC_50_ of PGD_2_ at the DP2 receptor sensor (S3 Fig). Our measurements at the DP1 receptor sensor using 10 µM PGD_2_ revealed faster activation (about twice as fast), and even faster deactivation (about nine times as fast) kinetics indicating different molecular mechanisms of ligand entry and binding in both receptors (Fig. 3D–H; 19.6 s ± 3.6; 0.8 s ± 0.1; mean ± SEM). Both the differences in activation kinetics as well as the differences in deactivation kinetics upon agonist withdrawal, proved to be significantly different between DP1 receptor and DP2 receptor (Fig. 3G,H).

### DP2 receptor sensor as a tool for the investigation of the characteristics of antagonists at DP2 receptor

**Figure 4:**
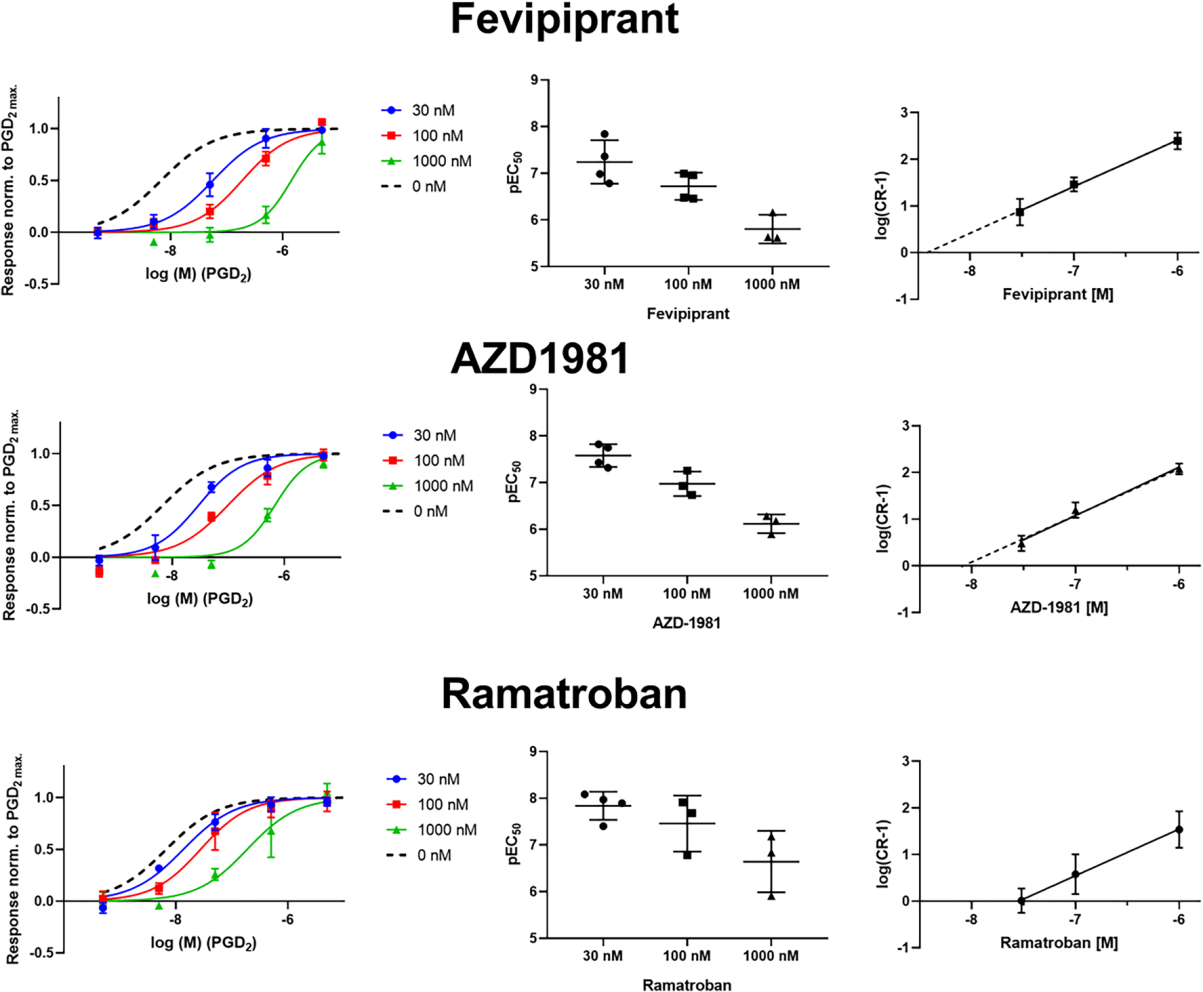
Characterization of antagonist effects on the DP2 receptor sensor. Effect of increasing concentrations of DP2 receptor antagonist (**A**) fevipripant (**B**) AZD1981 and (**C**) ramatroban on the PGD_2_-activated DP2 receptor sensor activity. Concentration response relationships of PGD_2_ at DP2 receptor were investigated in the presence of either 30 nM, 100 nM or 1 µM of the respective tested antagonist. We used the PGD_2_ concentration response curve from Fig. 1 as a reference (sigmoidal fit shown as dashed line). The middle panel shows pEC_50_ for the individual experiments. The pEC_50_-values were used to calculate concentration-ratios (CR) for PGD_2_. The right panel shows the double log plot of (CR-1)-values dependent on the concentration of the indicated antagonist. The solid line shows the fitted linear regression. The slope values were 0.9993 (95% CI 0.312–1.687) for Ramatroban, 0.9991 (95% CI 0.544–1.454) for Fevipripant and 1.039 (95% CI 0.718–1.360) for AZD1981. The dashed line shows the respective linear regression with a slope constrained to 1 (for illustration purposes only). Data are presented as mean ± SEM.

DP2 receptor has been identified as a target for the treatment of type 2 inflammation associated disease and numerous DP2 receptor inhibitors have been developed. We tested the effect of selected inhibitors on the PGD_2_-activated DP2 receptor sensor to test the usefulness of our sensor in this aspect. Concentration response relationships of PGD_2_ at DP2 receptor were investigated in presence of either 30 nM, 100 nM or 1 µM of the respective tested antagonist. As a reference we used the PGD_2_ concentration response curve measured in absence of antagonist from Fig. 1. The Schild plots for the tested antagonists ramatroban, AZD1981 and fevipiprant (Fig. 4) revealed a linear relationship of log (CR-1) on the antagonist concentration with Schild slopes close to unity indicating a competitive antagonism. The competitive antagonism is in line with the literature, Sykes, D. A. et al. for example^7^. The data was refit to a slope of 1.0 to derive pK_B_ values. The pK_B_ for ramatroban was at 7.44 (95% CI 7.04–7.81) which is in range of the reported literature pK_i_ values for ramatroban ranging from 7.22 to 8.40^7,8,19–21,26^. For AZD1981 and fevipiprant, pK_B_ was at 8.06 (95% CI 7.79–8.33) and 8.38 (95% CI 8.13–8.62) respectively, which is close to the literature with pK_B_ values of 7.98 and 8.54^7^.

## Discussion

In this work, we present a human DP2 receptor FRET-based conformation sensor, which shows a strong alteration in the emission ratio upon agonist stimulation allowing for measurements in single cells as well as in microtiter plates. We found that the DP2 receptor sensor exhibited wild-type receptor properties in respect to its agonist and antagonist profile and that it could be used for screening and characterization of ligands in respect to their binding kinetics, affinity and efficacy.

### DP2 receptor sensor resembles wt-receptor properties

A number of studies addressed the affinities of the DP2 receptor for PGD_2_, ramatroban and a broad range of further ligands. Unfortunately, the methods relevant for affinity determination varied strongly among the different studies resulting in a huge scattering of the retrieved values and only limited comparability. These variations include temperature (experiments at either 4 °C, “room temperature” or 37 °C), the presence or absence of 0.1% BSA/HSA (including the absence or presence of a mathematical correction for protein binding), the investigation on human, murine or rat DP2 receptor and the presence or absence of GTPγS^5,7,9,12,18–21^.

The majority of these studies reported pK_i_ values of 7.42–8.70 for PGD_2_ at DP2 receptor, which fits well to our observed pEC_50_ value of 8.20^5,9,12,18–21^, while there was one study reporting a pK_i_ value for PGD_2_ at DP2 of 6.40. This might possibly be explained by the presence of 30 µM GTPγS and the fact that the measurements were performed at 37 °C^7^.

The DP2 receptor sensor reassembled wt-receptor properties in terms of agonist selectivity. While substances, which are known to hardly bind to DP2 receptor, namely PGE_2_, U-46619 and iloprost, showed only minor activation of the DP2 receptor sensor at concentrations as high as 10 µM, known agonists such as DK PGD_2_, indomethacin, and 15(S)-15 methyl PGD_2_ showed activation of the DP2 receptor and could be used to measure concentration response curves. We ﻿determined the maximum effect of each compound by measuring concentration response curves normalized to the maximal effect of PGD2 (S2B Fig). ﻿Even though we used only five different concentrations of the tested compounds for the assessment of the concentration response curve, the pEC_50_ values fitted overall well within in the rather broad range of pK_i_ values reported in the literature^5,7,9,18,19^.

While recent understanding of the structures of DP2 receptor and other prostanoid receptors provided further insights into the binding modes of agonist and antagonist and the dissociation kinetics of antagonists from DP2 receptor gained intensive attention, not much is known about the dynamics of PGD_2_ induced DP2 receptor activation and deactivation^7,12,13,27,28^. We investigated PGD_2_ induced activation of DP2 receptor and DP1 receptor side-by-side and observed activation of the DP1 receptor twice as fast as compared to the DP2 receptor. In line with the differences in affinities, the wash-out kinetics were 9 times faster at the DP1 receptor compared to the DP2 receptor. There are two lipid recognition modes proposed for the prostanoid receptors, the “polar group in” mode for DP2 receptor and the “polar group out” mode for the remaining prostanoid receptors^12^. The charge distribution of the DP2 receptor is thought to guide the PGD_2_ to its final binding position via opposite charge attraction^12,27^. The differences in ligand entry between DP2 receptor and the rest of the prostanoid receptor family could potentially also be the cause of the observed differences in receptor activation and deactivation kinetics between DP1 and DP2 receptor using PGD_2_. Taken together, these experiments give further insight into the conformational dynamics of the DP1 receptor and DP2 receptor and highlight the suitability of our DP2 receptor sensor to resolve kinetics.

### DP2 receptor sensor as a suitable tool to investigate new DP2 receptor antagonists

DP2 receptor signaling could be linked to allergic inflammation and DP2 receptor antagonism has been identified as a strategy to treat allergic diseases in humans and many DP2 receptor antagonists have been investigated in this respect^6,7^. The development of refined DP2 receptor antagonists has also gained considerable interest beyond allergic disease and might extend to the treatment of diseases in the field of the central nervous system, in organs such as the lungs, intestines and kidneys and in cancer treatment^29^. Our evaluation of the suitability of our sensor for high throughput screenings resulted in a Z-factor value corresponding to an excellent assay^17^, (Fig. 1F; S1 Fig) potentially laying the groundwork for future high throughput antagonist screenings at the DP2 receptor. The Z-factor value is better than the value that we measured for our previously published FRET-based EP4 receptor conformation sensor^16^.

This claim is further supported by the fact that, our sensor was well suited to characterize established DP2 receptor antagonists. The observed affinities of DP2 receptor for AZD1981, fevipiprant and ramatroban matched well to the literature and the competitive binding mode was clearly visible in our measurements^7,8,19–21,26^. The initial characterization of AZD1981 to “not behaving as a simple competitive antagonist”^23^ could not be verified by our Schild plot analysis. The slope and estimated pK_B_ of our measurements at DP2 receptor sensor support the finding of competitive binding rather than the involvement of a non-competitive interaction component of AZD1981 at DP2 receptor and are in line with the results obtained by a Schild plot analysis based on [^35^S]-GTPγS binding assays by Sykes and colleagues^7^.

To sum up, we could show that our sensor was suited for detecting agonist efficacy, affinity and binding kinetics and proved to be useful in characterizing antagonists at the DP2 receptor. This is of substantial interest as blocking DP2 receptor activity is a promising strategy in diseases of the central nervous system, inner organs and in cancer treatment^29^.

## Materials and methods

### Plasmids and agonist

cDNA encoding the human DP1 receptor (Prostaglandin D_2_ Receptor (PTGDR), NM_000953, Catalog Number: #PTGDR00000) and the human DP2 receptor (Prostaglandin D_2_ receptor (CRTH2/GPR44), AY507142, Catalog Number: #CRTH200000) was obtained from the Missouri S&T cDNA Resource Center.

In this study we used U-46619 (16450), prostaglandin D_2_ (12010), prostaglandin E_2_ (14010), 8-iso prostaglandin E_2_ (14350), Prostaglandin E_1_ (13010), 8-iso prostaglandin E_1_ (13360), 8-iso prostaglandin F_2α_ (16350), Indomethacin (70270), 15(S)-15-methyl Prostaglandin D_2_ (12730), 13,14-dihydro-15-keto prostaglandin D_2_ (12610), Ramatroban (10156), AZD1981 (20763), Fevipiprant (20263) and Iloprost (18215) Manufacturer: Cayman Chemical, Ann Arbor, MI, USA.

The compounds were dissolved as stock solutions in either ethanol or DMSO. The stock solutions were further diluted in buffer containing 0.1% bovine serum albumin (Bovine serum albumin from heat shock fraction, protease free, fatty acid free, essentially globulin free, Sigma Aldrich, 9048-46-8).

### Cloning

The DP2 receptor sensor and DP1 receptor sensor were generated using the Q5® High-Fidelity DNA Polymerase and NEBuilder® HiFi DNA Assembly kit (New England Biolabs, Ipswich, USA) or using restriction enzymes followed by a subsequent ligation reaction using T4 ligase (New England Biolabs). All primers used for DNA amplification were designed in SnapGene Viewer (GSL Biotech LLC, Unites States).

### Generation of FRET-based DP2 receptor sensor

In a first step, we generated a DP2 receptor construct, with a mTurq2 fused to the C-terminus. To do so, we inserted the DP2 receptor, which was amplified with the following primers (fw: 5′- CGCAAATGGGCGGTAGGCGTG, rv: 5′ AAAAAATCTAGAACTCGAGGTGCTGCTCAGC), and subcloned into mTurq2 pcDNA3-backbone vector using HindIII and XbaI restriction sites.

Subsequently, an eYFP flanked with AscI and HpaI was inserted into the ICL3 of DP2 receptor- mTurq2 between position R240 and P241. To do so, eYFP was amplified as a fragment using: fw: 5′GGCGGGGGCGCGCCATGGTGAGCAAGGGCGAG and rv: 5′CTGGGTTAACCTTGTACAGCTCGTCCATGCC and DP2- mTurq2 was amplified as a vector using: fw: 5′GCTGTACAAGGTTAACCCAGGCCGCTTCGTGCGC, rv: 5′CACCATGGCGCGCCCCCGCCGGCGGCCG.

### Generation of FRET-based DP1 receptor sensor

DP1 receptor- mTurq2 was constructed by deleting amino acids of the C-terminus of DP1 receptor after position S357 and fusing mTurq2, flanked with AgeI and SacII to the truncated DP1 receptor. DP1 receptor was amplified as a vector with fw: 5′- GTAACCGCGGGAATTCTGCAGATATCCAGCACAGTG and rv: 5′-CCATACCGGTGGATTCCATGTTAGTGGAATTGCTGC and mTurq2 as a fragment was amplified from using the following primers: fw: 5′-GAATCCACCGGTATGGTGAGCAAGGGCGAG; rv: 5′-AATTCCCGCGGTTACTTGTACAGCTCGTCCATGCC. Subsequently we inserted an eYFP in ICL3 of DP1 receptor- mTurq2, flanked with AscI and HpaI, between T240 and E252. We amplified DP1 receptor- mTurq2 as a vector with fw: 5′-CAAGGTTAACGAAGCGTCCCCTCAGCC; rv: 5′-CATGGCGCGCCCGGTGCAGGAGCGCGG and eYFP as a fragment with fw: 5′-GGGCGCGCCATGGTGAGCAAGGGCGAG and rv: 5′-CGCTTCGTTAACCTTGTACAGCTCGTCCATGCC.

### Cell culture and transfection

For this study, transiently or stably transfected cells were used. HEK293T cells were cultured in Dulbecco's Modified Eagle's Medium (DMEM, 4.5 g L-1 glucose) containing 10% FBS, 2 mM L-glutamine, 100 U mL^-^1 penicillin and 0.1 mg mL^-^1 streptomycin at 37 °C in a humidified atmosphere with 5% CO_2_. DMEM, FBS, L-glutamine, penicillin and streptomycin were supplied by Capricorn Scientific GmbH (Ebsdorfergrund, Germany). To study the FRET-based DP2 receptor sensor, a stable cell line was generated by transfecting HEK293 cells with 1 µg FRET-based DP2 receptor sensor plasmid cDNA using Effectene reagent (Qiagen, Hilden, Germany) according to the manufacturer's instructions (in a 6 cm ∅ dish). Subsequently, the cells were cultured in DMEM medium containing G-418 (instead of penicillin and streptomycin), which is used as a selective agent. G-418 sulphate was purchased from Capricorn Scientific GmbH (Ebsdorfergrund, Germany).

All transient transfections were performed in HEK293T cells two days prior the measurement. **Transient transfections for FRET-based experiments** were performed analog to the procedure described for the stable transfection of HEK293 cells using the Effectene reagent with either 1 µg of DP2 receptor sensor or DP1 receptor sensor per 6 cm ∅ dish. Transfected cells were harvested the following day and seeded onto either sterile poly-L-lysine coated coverslips for measurements on the next day.

### Single cell FRET measurements

FRET signals of DP1 and DP2 receptor sensor were recorded from selected transient transfected single cells using an inverted microscope (Axiovert 100) and a previously described measurement setup (Jelinek et al., 2021). The cells were superfused with external buffer (137 mM NaCl, 5.4 mM KCl, 2 mM CaCl_2_, 1 mM MgCl_2_, 10 mM HEPES, pH 7.32) or external buffer containing ligand in the respective concentration by using a pressurized perfusion system (Fig. 3B and S3 Fig). The measurements were performed at room temperature and data was collected by the VisiView software (Visitron Systems). The data was corrected for background fluorescence, bleed-through and false excitation using Microsoft Excel. We referred this data as the eYFP/ mTurq2 emission ratio. A correction for photobleaching was performed using Origin 2017. This data was referred to as Δ(eYFP/ mTurq2). All single cell FRET measurements shown in this study were recorded at 1 Hz.

### FRET measurements of multiple cells in the plate reader

We used a Spark 20 M (Tecan) plate reader for FRET measurements on multiple cells. Greiner Bio-One μClear™ bottom 96-well polystyrene microplates were coated with poly-L-lysine. 100,000–120,000 transfected HEK cells were counted and seeded per well on the day before measurements. Initially, the culture medium was removed, and the cells were washed with external buffer. Subsequently 180 µl of external buffer was added into each well and 20 µl external buffer or external buffer with ligand solution was used for each application. The measurements were controlled using the software SparkControl™ and were performed as fluorescence bottom readings using filters: mTurq2 was excited at 430 nm and the emitted donor fluorescence (mTurq2) and acceptor fluorescence (eYFP) were recorded at 485 nm and 535 nm respectively. All experiments, with the exception of the Z-factor value measurements, were measured at least in technical duplicates. After each measurement, the mTurq2 and eYFP emission were exported to Microsoft Excel and the (eYFP/mTurq2) emission ratio was calculated. Buffer wells were used as control and subtracted. This data was referred to as Δ(eYFP/mTurq2). In all measurements except for the Z-factor value measurements in each cycle first mTurq2 was excited and the mTurq2 emission was recorded for all measured wells, followed by the excitation of mTurq2 and the recording of eYFP emission for all measured wells (measurement “whole plate mTurq2 than whole plate eYFP”). In case of the Z-factor value measurements in each cycle every individual well was first excited at 430 nm and the emitted donor fluorescence (mTurq2) was recorded followed by a second excitation at 430 nm and the recording of the acceptor fluorescence (eYFP; measurement “well by well”).

### Analysis of concentration–response relationships

Concentration–response relationships were evaluated for PGD_2_ at DP1 receptor sensor in single cell FRET measurements (S3 Fig). Single cells were superfused with the tested concentrations followed by a reference concentration of 5 µM PGD_2_. Each agonist induced amplitude was normalized to the amplitude of the respective reference concentration. Concentration–response relationships were evaluated for PGD_2_ (Fig. 1), Indomethacin, 15(S)-15-methyl prostaglandin D_2_, 13,14-dihydro-15-keto prostaglandin D_2_ (Fig. 2) and prostaglandin D_2_ in presence of indicated antagonist concentrations (Fig. 4) in multiple cell FRET measurements at the Tecan plate reader. Multiple cells were measured in a 96 well format. In each measurement one test concentration of the investigated compound and a reference concentration of PGD_2_ was applied except for the PGD_2_ measurements in presence of indicated antagonist concentrations. The responses of the tested concentration were evaluated relative to the response of the reference concentration of PGD_2_. The reference concentration of PGD_2_ in multiple cell FRET measurements was a final concentration of 10 µM PGD_2_. In case of the PGD_2_ measurements in presence of antagonist, the saturating agonist concentration was applied to independent wells to assure a maximal response of the reference. In some instances, due to technical reasons these independent wells contained neglectable concentrations of the tested antagonist. All concentration–response curves were fitted with GraphPad Prism 8. The concentration response curves used for fitting pEC_50_ values presented in Figs. 1 and 2 were fitted using log(agonist) vs. response – (three parameters) with variable EC_50_ while bottom was constrained to 0 and top was constrained to be less than 1.01.

The following Eq. 1 was used for fitting:

1 $$Y = Bottom + \frac{Top - Bottom}{{1 + 10^{{\left( {Log EC50 - X} \right)}} }}$$

The concentration response curves underlying Fig S3 and Fig. 4 were fitted using log(agonist) vs. response – Variable slope (four parameters) with variable EC_50_ while bottom (0) and top (1) were constrained.

The following Eq. 2 was used for fitting:

2 $$Y = Bottom + \frac{Top - Bottom}{{1 + 10^{{\left( {Log EC50 - X} \right) \times Hillslope^{{}} }} }}$$

Kinetics of receptor activation upon agonist application and receptor deactivation upon agonist withdrawal were determined by fitting the inverted FRET response of DP1 receptor sensor and DP2 receptor sensor (Fig. 3) to a mono-exponential function. In case of receptor activation, Y_0_ was constrained to 0. Curve-fitting and calculation of respective halftime values were performed using GraphPad Prism 8.

### Calculation of Z-factor values

For comparison reasons Z-factor values were calculated as described in Schihada et al*.* 2018 using the term Eq. 3, which is based on the formula provided by Zhang et al.^17,30^:

3 $${\text{Z}} - {\text{factor value }} = { 1 } - \frac{3SD c + 3SD s}{{average c - average s}}$$

where SD and average are the SDs and average ΔFRET [%] values of 1 µM PGD_2_ (sample, **s**) and external buffer control (control, c), respectively. Control wells are shown in black (buffer application) and sample wells in red (PGD_2_ application).

### Schild plot analysis

The pEC_50_-values shown in Fig. 4 were divided by the pEC_50_ of PGD_2_ in absence of antagonist shown in Fig. 1 in order to obtain concentration-ratios (CR) values. The double log plot of (CR-1)-values dependent on the concentration of the indicated antagonist were plotted and a linear regression was fitted. Subsequently to obtain pk_B_ values the averaged concentration response curves were fitted with GraphPad Prism 8 to the Gaddum/Schild equation with Schild slope constrained to 1, and constrained top and bottom.

### Statistical analysis

The data shown in this study is either presented as single example measurements or as the mean ± SEM of the stated number of independent experiments. Graphpad Prism 8 was used for statistical analysis. Datasets presented in S2A Fig were analyzed by one sample t test against a theoretical mean of 0. Datasets presented in S2B Fig were analyzed by one a one-way ANOVA test. Datasets presented in Fig. 3 for activation and deactivation kinetics were analyzed with an F-test to compare variances. Based on the outcome the datasets were subsequently analyzed for activation kinetics using an unpaired t-test, while the kinetics of receptor deactivation kinetics were compared using an unpaired t-test with Welch's correction. Differences were considered statistically significant for *P* ≤ 0.05.

## Supplementary Information

Supplementary Information.

## Data Availability

All the data generated in the study are included in the manuscript and supplemental figures. The raw and processed data supporting the findings of this study are available on reasonable request from the corresponding author.
