# Supplementary material for: DP2 receptor activity sensor suited for antagonist screening and measurement of receptor dynamics in real-time
Source: Sci Rep. 2024 Apr 8;14:8178. doi: 10.1038/s41598-024-58410-2 (PMC11374897; doi:10.1038/s41598-024-58410-2)
Supplement: Supplementary file 1 — Supplementary Information. [file 41598_2024_58410_MOESM1_ESM.pdf]

DP2 receptor activity sensor suited for antagonist screening and measurement of receptor dynamics in real-time

Supplemental figures

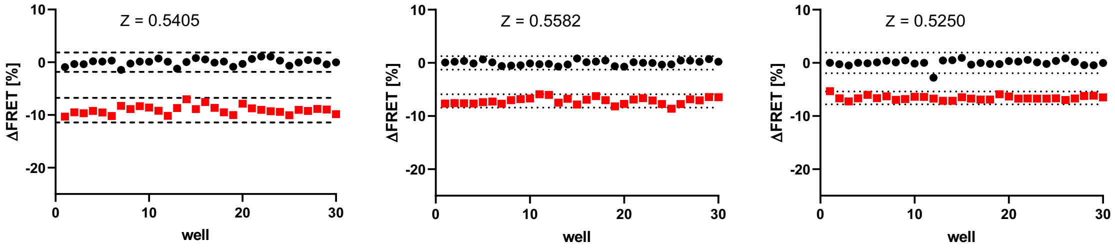

S1 Fig Determination of the Z-factor value of the DP2 receptor sensor. HEK293 cells stably expressing DP2 receptor sensor were seeded in three different 96 well microplates. The microplates were measured in the plate reader described in Fig 1A. Control wells are shown in black (buffer application) and sample wells in red (PGD_2 application). Changes in Δ(eYFP/mTurq2) in percent are plotted for each well. Each point of each data set was subtracted by the mean of the control data set. Control wells (buffer) are shown in black, sample (final: 1 µM PGD_2) are shown in red. Broken lines display three standard deviations (SD) from the mean of each data set. Each graph represents one 96 well plate, which results in 30 control and 30 sample wells as outer wells were excluded from the evaluation.

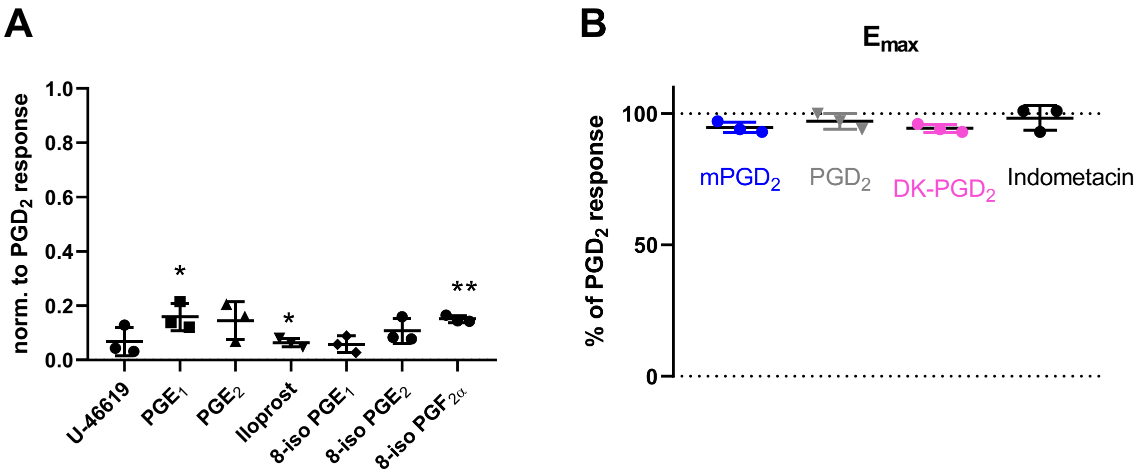

S2 Fig Tested prostanoids with minor effects on DP2 receptor sensor activity and Emax values of selected compounds on DP2 receptor sensor activity compared to PGD_2. A) A final concentration of 10 µM, except for 8-iso $PGF_{2\alpha}$ where 5 µM were used, of the substances indicated in the figure legend was tested on their ability to evoke alterations in the emission ratio at the DP2 receptor sensor using a similar protocol as shown in Fig 2. The amplitude evoked by the tested substance was compared to the amplitude evoked by a subsequent application of a final concentration of 10 µM PGD_2 within the same well. Data are shown as mean ± SEM. P(PGE_1) = 0.0317*, P(Iloprost) = 0.0193*, P(8-iso $PGF_{2\alpha}$) = 0.0024** P(other)>0.05 as determined by one sample t test against a theoretical mean of 0. B) The panels shows E_{max} for the individual experiments performed for each ligand presented in Fig 2. $mPGD_2$ 95 % ± 1%, PGD_2 97% ± 2%, DK- PGD_2 94% ± 1% and Indomethacin 98% ± 3%. Data are shown as mean ± SEM, one-way ANOVA (P = 0.3745).

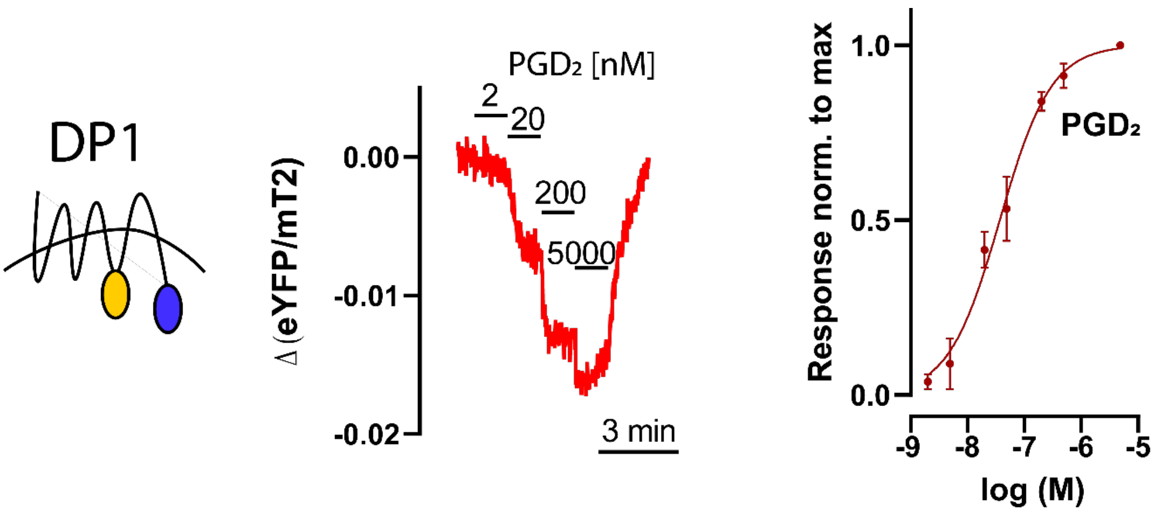

S3 Fig. Concentration response curve at DP1 receptor sensor with PGD_2. HEK293T cells were transiently transfected with DP1 receptor sensor. Left: The panel shows the emission ratio Δ(eYFP/mTurq2) of a representative trace (out of n=5), corrected for photobleaching and superfused with the indicated concentrations of PGD_2 overt time. Right: Concentration response curve of measurements as shown (Left) and analog. Each agonist induced amplitude was normalized to the amplitude of a reference concentration of 5 µM PGD_2, which was included in each measurement. pEC_{50} 7.43 (95% confidence interval: 7.49 to 7.37) n = 5-6 per data point, mean ± SEM.
